# Youth physical activity and the COVID-19 pandemic: A systematic review

**DOI:** 10.1016/j.pmedr.2022.101959

**Published:** 2022-08-22

**Authors:** Bridgette Do, Chelsey Kirkland, Gina M. Besenyi, Carissa Smock, Kevin Lanza

**Affiliations:** a Department of Population and Public Health Sciences, University of Southern California, Los Angeles, CA 90032, USA; b Center for Public Health Systems, School of Public Health, University of Minnesota, Minneapolis, MN 55455, USA; c College of Public Health, Kent State University, Kent, OH 44240, USA; d Department of Kinesiology, College of Health and Human Sciences, Kansas State University, Manhattan, KS 66506, USA; e School of Business, Northcentral University, San Diego, CA 92123, USA; f School of Public Health in Austin, University of Texas Health Science Center at Houston, Austin, TX 78701, USA

**Keywords:** Exercise, COVID-19, Adolescents, Children, (PA), physical activity, (COVID-19), coronavirus disease 2019

## Abstract

The purpose of the systematic review was to identify, evaluate, and synthesize evidence from available published literature examining the impact of the coronavirus disease 2019 (COVID-19) pandemic on youth physical activity (PA). A systematic review of the literature was conducted for years 2020–2021. Published articles were searched in eight databases. Inclusion criteria included: availability of full-text, written in English language, and reported quantitative or qualitative results of original or secondary data on PA and COVID-19 related factors among youth (ages 5–17 years). A standard quality assessment tool assessed risk of bias and quality of included articles. The search retrieved 2,899 articles with 51 articles ultimately meeting inclusion criteria. The majority of articles (65 %) investigated change in PA from before to during the pandemic. Most evidence indicated an overall decrease in youth PA levels during the pandemic with differences observed among sub-populations (e.g., age, sex or gender), type, and location. Findings suggest pandemic-related closures hindered PA participation due to a high reliance on school- and sport-based PA. Programmatic strategies (e.g., activity breaks, active curriculum, free online activities/lessons) should include aligning intervention measures and geared towards evolving and ongoing PA promotion based on the latest findings.

## Highlights

• Numerous studies examined youth physical activity (PA) during the COVID-19 pandemic.
• Overall decreases in youth PA were seen during the first 1.5 years of the pandemic.
• There were differences in PA by sub-populations (e.g., age, sex/gender)
• There were also differences in PA by location/type (e.g., outdoor, play)
• Programming and policy should focus on evolving PA promotion for youth.

## 1 Introduction

Physical activity (PA) is an essential behavior for child and adolescent health. Youth who regularly engage in PA have improved cardiorespiratory fitness, bone and muscle growth, weight control, and reduced symptoms of anxiety and depression compared to less active peers. (Piercy et al., 2018) Beyond health benefits, PA improves youth’s academic performance. (Singh et al., 2019) While PA engagement has wide-ranging benefits for individuals across all life stages, (Institute, 2018) PA during childhood and adolescence is critical since it tracks into adulthood (Hayes et al., 2019) and consequently reduces the risk of all-cause mortality, cardiovascular disease, type II diabetes, and several cancers. (Piercy et al., 2018) Prior to the onset of the World Health Organization (WHO) declaring coronavirus disease 2019 (COVID-19) a pandemic in March 2020, (CDC. CDC Museum COVID-19 Timeline. Centers for Disease Control and Prevention. Published August 4, 2021) youth already did not obtain recommended levels of PA; globally, 81 % of youth aged 11–17 years did not meet the 2010 WHO recommendation of at least 60 min of moderate-to-vigorous PA (MVPA) daily, in 2016. (World Health Organization. Physical activity. Published November 26, 2020).

In addition to resulting in over 5.41 million deaths (as of December 2021), (Johns Hopkins University. COVID-19 Dashboard. Johns Hopkins Coronavirus Resource Center. Accessed December 7, 2021) the COVID-19 pandemic has dramatically altered everyday life and taken a significant toll on the mental health and well-being of individuals, especially youth and caregivers. (Panda et al., 20212021) To prevent or reduce local COVID-19 transmission, individuals and communities adopted strategies comprising personal controls, administrative controls, and engineering controls. (CDC. Mitigation Measures for COVID-19 in Households and Markets in Non-US Low-Resource Settings. Centers for Disease Control and Prevention. Published February 11, 2020) These preventive measures varied substantially by region and date of implementation, level of stringency, and degree of adherence. (Petherick et al., 2021, Hale et al., 2021, Sorci et al., 2020) Youth PA is significantly impacted by these preventive strategies, especially school closures, since youth spend a large portion of waking hours at school where there are ample opportunities for PA such as physical education (PE) class, recess, sports, and active transport to and from school. (Szeszulski et al., 2021) Changes to daily routine due to COVID likely negatively impacted youth mental health. (Panda et al., 20212021) These daily routine changes coupled with additional pandemic mental health burden may have also altered youth PA patterns compared to before the pandemic. (Piercy et al., 2018).

A previous systematic review on changes in PA behavior from before to during the COVID-19 pandemic identified 66 articles focusing on ages 13–86 years, wherein the majority reported decreases in PA across reviewed populations during the pandemic. (Stockwell et al., 2021) Half of the included articles used unvalidated questionnaires; the systematic review was limited to articles published from November 2019 to October 2020; and results were not disaggregated to make conclusions on youth PA. Another review of articles conducted between March 2020 and April 2021 on youth PA identified a decrease in PA during the pandemic ranging 10.8–91 min per day, with limited increases in PA related to unstructured and outdoor activities. (Rossi et al., 2021) This evidence synthesis on youth PA was limited by its exploratory nature as a scoping review, (Rossi et al., 2021) a preliminary assessment that can serve as a helpful precursor to a more resource intensive systematic literature review. (Munn et al., 2018) In addition, a systematic review protocol has been proposed for measuring the effect of COVID-19 on PA among school-aged youth (6–17 years), but has not been undertaken prior to our review. (Hu et al., 2021) Although previous studies have assessed PA during the COVID-19 pandemic, a systematic review among youth (ages 5–17) is critical to delineate PA changes among this population to inform researchers, practitioners, and decision makers.

Therefore, the overall objective of this systematic review was to identify, evaluate, and summarize the available literature through May 10, 2021, on the impact of the COVID-19 pandemic on youth PA. Specific aims of the review were to determine PA levels of youth during the pandemic, investigate whether the pandemic was associated with a change in youth PA, and evaluate differences in youth PA by type, location, age, and sex or gender. The review also identified available evidence and described the timeframe, setting, sample, design, and results of empirical studies assessing youth PA levels during the pandemic and whether youth PA levels changed compared to before the pandemic. Determining how the pandemic influenced youth PA can inform the design of PA interventions, benefiting a protective health behavior during and after the COVID-19 pandemic and other infectious disease outbreaks. (Sallis et al., 2020).

## 2 Methods

The authors include a diverse range of academic and professional disciplines (public health, kinesiology, urban planning, parks and recreation) with published and nationally presented research in PA, have obtained a doctoral degree or are doctoral candidates, and hold leadership positions within the American Public Health Association (APHA) Physical Activity Section. These leaders met monthly to develop, conduct, and discuss the review. The mission of the PA Section is “to provide leadership and to advocate for policies, practices, environmental changes, and research that support physical activity” (https://www.apha.org/APHA-Communities/Member-Sections/Physical-Activity). This systematic literature review will summarize the effects of the COVID-19 pandemic on youth PA and provide a foundation for the development and dissemination of evidence-based programs and policies.

**Fig. 1:**
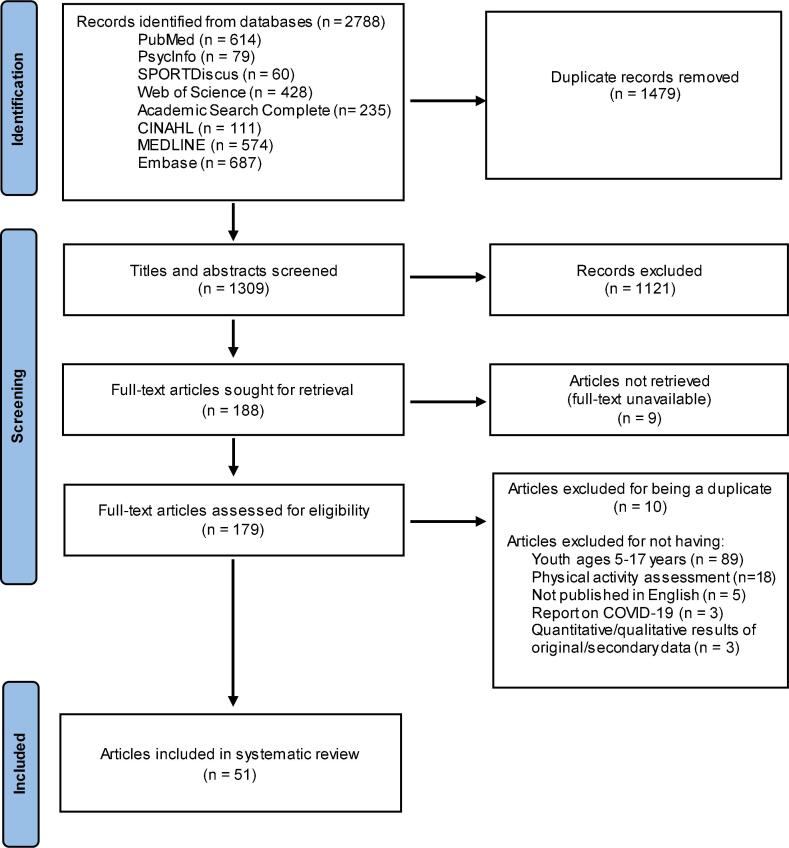
PRISMA Flow Diagram of Article Selection.

The protocol was registered online in Open Science Framework (OSF), as a systematic review. Guided by the 2021 Preferred Reporting Items for Systematic Reviews and meta-Analyses (PRISMA) (Page et al.,), searches in PubMed, PsycInfo, SPORTDiscus, Web of Science, Academic Search Complete, CINAHL, MEDLINE, and Embase databases were conducted across all dates through May 10, 2021. The review focused on January 2020 through May 2021, a time frame thought sufficient to capture the changes in COVID-19 cases, deaths, and regulatory and behavioral responses, and ultimately, to address the review objectives. The following string was searched within each database: (‘physical activity’ OR ‘exercise’ OR ‘sport’) AND (‘COVID-19′ OR ‘coronavirus’ OR ‘SARS-CoV-2′) AND (‘youth’ OR ‘child’ OR ‘adolescent’ OR ‘teen’). Inclusion criteria for targeted articles included: (1) reported on PA (e.g., frequency, duration, intensity, type, location); (2) reported on how COVID-19 impacted PA; (3) study population was youth (5–17 years); (4) reported quantitative or qualitative results of original or secondary data; (5) human subjects research; (6) published in English language; and (7) full-text article was available. All records were imported into Covidence systematic review software (Veritas Health Innovation, Melbourne, Australia; https://www.covidence.org). All articles were screened by authors BD and CK to avoid errors. Titles and abstracts were first screened for inclusion with disagreements resolved via consensus. Full text was obtained for potentially eligible articles and assessed for inclusion. Articles were further excluded if (1) no quantitative or qualitative data were provided (e.g., commentaries, protocols); (2) did not examine PA in the context of the COVID-19 pandemic (e.g., data collection was during the pandemic, but the potential effects of the pandemic were not considered); and (3) participants aged < 5 or 18 + were not disaggregated by age allowing results for participants within the age range of interest of this study to be obtained. Discrepancies were discussed and resolved by authors BD and CK. Details on paper selection were illustrated in a PRISMA diagram (Fig. 1).

Authors BD and CK extracted data from each eligible article: title, author; study country; study objective; study design; data collection timeframe; recruitment; sample size; youth characteristics; parent/caregiver characteristics; who reported data (e.g., child or parent/caregiver); COVID-19 context specifics (e.g., local restrictions); COVID-19 factors examined (e.g., school closures, home confinements); whether pre-pandemic data were used; PA measurement; PA report timeframe (e.g., past week); PA outcome and findings; and limitations.

Authors BD and CK used the “standard quality assessment criteria for evaluating primary research papers from a variety of fields” tool (Kmet et al., xxx)to score risk of bias and quality of included articles, per a previous systematic review. (Van Hecke et al., 2018) The tool consists of a 10-item qualitative checklist and 14-item quantitative checklist, both of which were inputted in Covidence for scoring. Quantitative articles were scored depending on the degree to which criteria were met (“yes” = 2, “partial” = 1, “no” = 0, or “n/a” if not applicable). We calculated a summary score for each article by summing the total score across relevant items and then dividing by the maximum score (20 for qualitative studies, 28 for quantitative studies, 48 for mixed-methods studies) excluding “n/a” items. Interrater reliability between the two coders was deemed excellent (intraclass correlation coefficient = 0.98). (Koo and Li, 2016) All disagreements were discussed until a final score for each article was reached.

## 3 Results

Our search resulted in 2,899 articles from selected database sources based on inclusion criteria and search terms. After removing duplicates (n = 1,479), the remaining articles (n = 1,309) were screened by title and abstract and 1,121 were excluded. The full-text of the remaining 188 articles were reviewed to identify articles meeting inclusion criteria. Ultimately, the systematic review included 51 articles. Each article was reviewed in detail, and study characteristics and results were summarized (Table 1, Table 2). The mean quality score of all included articles was 0.89 (*SD* = 0.07; Range: 0.65–1.0).

**Table 1:** Characteristics and Main Findings of Included Studies that Assessed Current PA During the Pandemic (N = 18).

Country	Data Collection Period^a^	Type of Study	Sample^b,c^	Physical Activity Data	Main Findings	Study
Australia	May 4–31, 2020	Cross-sectional	963 adolescents ages 13–17 years	Self-report by youth	7 % of adolescents met the WHO PA guidelines. 28.1 % of adolescents met the WHO PA (muscle-strengthening) guidelines (higher than the national average in the past). 26.5 % used online or digital platforms for PA.	(Parker et al., 2021)
Canada	April 8–30, 2020	Cross-sectional	895 adolescents ages 12–17 years	Self-report by youth	Boys and girls did not differ in their sport habits. More time spent playing sports was associated with lower levels of positive affect.	(Tardif-Grenier et al., 2021)^47^
Chin	February 25-March 8, 2020	Cross-sectional	1,264 children ages 7–12 years	Self-report by youth	41.8 % did>2 days/week of > 60 min of PA. Children who regularly had PA during the pandemic were less likely to have hyperactivity-inattention and prosocial behavior problems.	Q. Liu et al. (2021)
China	March 8–15, 2020	Cross-sectional	4,898 adolescents	Self-report by youth	Average daily MVPA: 23.4 ± 52.5 min Boys had higher average MVPA minutes than girls. Boys accumulated more vigorous PA than girls, while girls had more moderate PA.	(Kang et al., 2021)
China	March 8–15, 2020	Cross-sectional	9,997 children and adolescents	Self-report by youth	Average MET-min/week: 1,193.02 ± 1,621.88Vigorous PA accounted for the largest proportion (42.74 %) while walking PA accounted for the lowest proportion (24.19 %). Authors concluded that PA declined during this period.	(Zhang et al., 2020)
China	March 8–30, 2020	Cross-sectional	1,199,320 children	Self-report by youth	Time spent exercising were associated with protection from adverse mental health: Students who spent<0.5 h exercising also had higher odds of self-reported psychological distress (OR: 1.64).	(Qin et al., 2021)
China	March 2020	Cross-sectional	17,029 children ages 6–12 years	Self-report by parent/caregiver	Average daily PA: 0.96 ± 0.84 h	Tso et al. (2020)
China	April 19–26, 2020	Cross-sectional	1,487 adolescents ages 10–17 years	Self-report by youth	PA time did not significantly differ between adolescents living in communities with vs without COVID cases.PA time was negatively associated with depressive symptoms.	(Ren et al., 2021)
China	April 2020	Cross-sectional	1,680 adolescents	Self-report by youth	69.5 % (72.4 % males, 66.4 % females) of participants did at least 150 min/week of PA. Male students had higher PA scores than female students. Students in lower grades were also more physically active than those in higher grades. Most frequently reported activities: indoor stretch, indoor resistance training, outdoor walking, jump rope, indoor run, outdoor run.	(Xiao et al., 2021)
China	May 12–18, 2020	Cross-sectional	3,405 children	Self-report by youth	For outdoor exercise, 620 (18.2 %), 398 (11.7 %), 1583 (46.5 %), and 804 (23.6 %) reported as frequent, somewhat frequent, somewhat infrequent, and infrequent, respectively. About 25 % did not perform any outdoor exercise during the school closures, and>70 % engaged in outdoor exercise less than three times per week.	J. Liu et al. (2021)
China	May13-20, 2020	Cross-sectional	965 adolescents ages 15–17 years	Self-report by youth	49.9 % of participants did<1.5 h/day of PA. Adolescents with high PA time reported less opportunities to develop insomnia symptoms and depressive symptoms than those with low PA time.	(Lu et al., 2020)
China	Not stated	Cross-sectional	192 children with ADHD	Self-report (unknown reporter)	Children with ADHD in the problematic digital media use group spent less days on physical exercise.	(Shuai et al., 2021)
Greece	November 2020-January 2021	Cross-sectional	950 adolescents ages 12–17 years	Self-report by youth	MVPA was below the 50 % of the recommended by the WHO PA for adolescents. Boys scored higher in PA compared to girls. Adolescents who used to participate in organized sport reported higher levels of PA and better well-being compared to those not participating in organized sport. Total PA was associated with well-being in model with sex, BMI, sedentary time, and eating behavior.	(Morres et al., 2021)
Italy	March 11–30, 2020	Cross-sectional	25 adolescents ages 13–17 years	Self-report by parent/caregiver	Moderate PA during the pandemic was lower in teenagers (compared to children under 12 years and adults over 18 years), even though the global mean daily PA was not different among groups.	(Di Dalmazi et al., 2020)
Portugal	March 23-April 1, 2020	Cross-sectional	1,091 children ages 6–12 years	Self-report by parent/caregiver	Boys and girls did not differ daily PA in either age group.No differences in 3 typologies of outdoor space (no outdoor space, small outdoor space, large outdoor space) children ages 10–12 years.	(Pombo et al., 2020)
Spain	May 2020	Mixed-methods cross-sectional	234 children ages 8–17 years	Self-report by parent/caregiver	162 % of parents said their child’s PA was insufficient. Qualitative results suggest that PA was conditioned by the type of housing: when there is no outdoor space to develop PA, parents find that their child’s PA is more insufficient.	López-Aymes et al. (2021)
U.S.	April 20-June 26, 2020	Cross-sectional	38 children exposed to gestational diabetes & 27 unexposed ages 9–15 years	Self-report by youth and parent/caregiver	Median MVPA and vigorous PA as lower among exposed to gestational diabetes, suggesting that engaging in vigorous PA during stressful periods was associated with reduced anxiety levels.	Alves et al. (2021a)
U.S.	April 22-July 29, 2020	Cross-sectional	64 overweight and healthy weight children ages 9–15 years	Self-report by youth and parent/caregiver	MVPA was unrelated to state anxiety.	Alves et al. (2021b)

^a^Dates are listed as shown in the full-text article; some articles did not specify dates.

^b^There was not a defined age range for “children” or “adolescents”; whichever term(s) the authors used is listed.

^c^Age ranges are not listed if the information was not available in the full-text article.

*Note.* Articles are sorted alphabetically by country and chronologically by date of data collection.

**Table 2:** Characteristics and Main Findings of Included Studies that Assessed Change in Physical Activity (PA) during versus before COVID-19 Pandemic (N = 33).

Country	Data Collection Period^a^	Type of Study	Sample^b,c^	PA Data	Direction of PA Change^d^	Main Findings	Study
Australia	May 2020	Cross-sectional	213 children ages 5–17 years with ADHD	Self-report by parent/caregiver	↓	Parents reported children did less regular exercise compared to 3 months prior. 50.8 % did < 5 days of exercise per week before the pandemic whereas 72.6 % did < 5 days of exercise per week during the pandemic. 50.3 % reported no change/positive change in PA vs 49.7 % reported negative change in PA.	Sciberras et al. (2020)
Australia	May 15-June 15, 2020	Mixed-methods; cross-sectional	157 children ages 5–9 years	Self-report by parent/caregiver	↕	Overall, weekly minutes of total PA did not change from before to during the pandemic. Frequency and duration of unstructured PA increased, whereas organized PA decreased. Outdoor play in the yard or street around the house, outdoor play in the park or playground or outdoor recreation area, and active indoor play at home all increased.Parents reported their children were more physically active during the pandemic due to: more children playing with each other in local streets, increased use of neighborhood spaces (e.g., parks) for PA, increased unstructured physical activities as a result of organized sport stopping.	(Nathan et al., 2021)
Brazil	March 25-April 24, 2020	Cross-sectional	423 children ages 6–12 years	Self-report by parent/caregiver	↓	Children ages 6–9 years spent more time playing without PA than children ages 10–12 years; however, there were not differences in playing time with PA or total time in PA between the two groups.	de Sá et al. (2020)
Brazil	June 12-July 12, 2020	Cross-sectional	426 children and adolescents	Self-report by youth	↓	PA decreased.	(de Matos et al., 2020)
Canada	1 month after WHO declared pandemic	Case-Control	1,472 children ages 5–7 years	Self-report by parent/caregiver	↕	The adherence to PA group included children whose parents reported that their children either maintained or increased (responded about the same, a little more, or a lot more) time spent walking/biking in their neighborhood.The non-adherence group included children whose parents reported a large decrease (responded ‘a lot less’) in their children’s outdoor PA/sport since COVID-19.	(Guerrero et al., 2020)
Canada	1 month after WHO declared pandemic	Cross-sectional	1,472 children ages 5–17 years	Self-report by parent/caregiver	↓	18.21 % of sample was currently meeting Canadian PA guidelines. There was a decline in all physical activities except household chores. The most dramatic decline was with outdoor PA and sport. Being a younger parent was associated with less decline in child PA, outdoor, and indoor play, and family PA.Parental encouragement was associated with higher child outdoor PA, time spent walking and biking, outdoor play, indoor PA, and family PA	(Moore et al., 2020)
Canada	January-April 2019 and 2020	Cross-sectional	109 children with congenital heart disease	Fibit Charge 2	↓	Children with congenital heart disease had a decline of 21 %-24 % of their overall daily step counts. Steps counts in the last week of March and first week were lower in 2020 than in matched weeks in 2019.	(Hemphill et al., 2020)
Canada	April 2020	Cross-sectional	1,472 children ages 5–17 years	Self-report by parent/caregiver	↕	26.5 % of those who increased outdoor activities met the guidelines vs 11.6 % of those who decreased outdoor activities met MVPA guidelines. 53 % reported less walking or biking, 64 % less outdoor PA or sport, 51 % less outdoor play, 53 % increase indoor play. More youth aged 12–17 (than children aged 5–11) experienced a decrease in PA-related movements during the pandemic. Those living in high-density neighborhoods were more likely to be in decrease outdoor activity.	(Mitra et al., 2020)
Canada	June 2–9, 2020	Cohort	20 student athletes ages 15–17 years	Self-report by youth	↕	PA declined at the onset of restrictions and at the onset of school resuming online. Poor weather (snow and ice) limited students to indoor exercises. At the time of interview, some participants returned to activity levels prior to the pandemic.	(Shepherd et al., 2021)
Canada	June-July 2020	Qualitative cross-sectional	29 children ages 5–11 years	Self-report by parent/caregiver	↓	Families saw a dramatic decline in children's PA. Major pandemic-related changes: loss of structured activities at school and afterschool, limited access to destinations (e.g., parks), barriers (e.g., weather, motivation). Parents said online and fitness-based activities were uninteresting for children and didn't provide same intensity of activity children are used to.	(Riazi et al., 2021)
Canada	September-December 2020	Qualitative cross-sectional	22 children ages 8–12 years	Self-report by youth and parent/caregiver	↑	There as an increase in unstructured play due to: closed structures and places to be active, less supervision, more time outside, reduced opportunities for structured activity. There was lost in play time to accommodate safety rules and sanitation protocols.	(Pelletier et al., 2021)
China	January 3–21, 2020 and March 13–23, 2020	Cohort	2,426 children and adolescents ages 6–17 years	Self-report by youth	↓	Average decrease in PA: 435 min/week Prevalence of physically inactive youth increased from 21.3 % to 65.6 %.	(Xiang et al., 2020)
China	March 5–15, 2020	Cross-sectional	10,416 children and adolescents	Self-report by youth or parent/caregiver	↓	Prevalence of youth who reported a decrease in time spent in PA was highest among boys. Prevalence of youth who reported a decrease in time spent in PA was highest among high school students.	(Guo et al., 2021)
China	July 2020	Cross-sectional	2,863 adolescents ages 9–17 years	Self-report by youth	↕	More participants reported decrease time to exercise (33.3 %) than those who reported increase time to exercise (25 %). No male/female differences in time spent to exercise. More primary school students reported increased time to exercise than secondary school students. More students from the high affluence group reported increase time to exercise than the low affluence group.	(Zhu et al., 2021)
Cyprus	June 1-July 17, 2020	Cross-sectional	1,509 children	Self-report by parent/caregiver	↓	Following the re-opening of schools, children’s PA decreased both in school and in their free time compared to before the pandemic. Children’s PA was reduced during the pandemic.	(Konstantinou et al., 2021)
Cyprus & Greece	February 3-April 26, 2019 and 2020	Cohort	108 children with asthma	EMBRACE smartwatch	↓	There was a steep decrease in total steps per day.	(Kouis et al., 2021)
Czechia	June 2020	Qualitative cross-sectional	3,440 adolescents	Self-report by youth	↕	Boys who reported daily MVPA (before pandemic) were 2.8 times more likely to perceive they did more MVPA during the Spring of 2020 lockdown, and 1.7 times more than girls in the same group.	(Ng et al., 2021)
France	April 1-May 6, 2020	Cross-sectional	6,491 children and adolescents ages 6–17 years	Self-report by youth or parent/caregiver	↓	Children who reported doing < 2 h or > 5 h 30 min of PA per week before lockdown increased this practice time during the confinement period. Conversely, the proportion of children who practiced between 2 h and 5 h 30 min of PA per week reduced their practice during confinement. 48.9 % of the children who practiced < 5 h 30 min of PA per week before confinement increased their duration of practice during the confinement period.In general, 42.0 % of children decreased their level of PA, while it remained stable in 21.3 % and increased in 36.7 %. On average, 58.7 % of adolescents decreased their level of PA, 21.8 % did not change, and only 19.6 % increased.	(Genin et al., 2021)
France	April 1-May 6, 2020	Cross-sectional	6,491 children and adolescents ages 6–17 years	Self-report by youth or parent/caregiver	↕	42 % children & 58.7 % adolescents reported decreased PA during the pandemic. 21.3 % children & 21.8 % adolescents maintained similar PA. 36.7 % children & 19.6 % adolescents increased PA. Being physically active before the pandemic was associated with a change in PA level during the pandemic such that initially active children and adolescents decreased their PA more than those initially inactive.Geographic location (urban, suburban, rural) was associated with PA. 64.2 % reported a decrease in PA when they did not have access to an outdoor area, compared to a decrease in PA among only 37.8 % who had individual outdoor area access.	(Chambonniere et al., 2021)
Germany	June 11–19, 2020	Cross-sectional	193 children ages 8–13 years	Self-report by youth	↓	A reduction in PA was only seen among primary school students (42.2.%).	(Hommes et al., 2021)
Israel	December 4–10, 2020	Cross-sectional	382 children	Self-report by parent/caregiver	↓	Children spent less time in PA. There was a significant interaction of time-point (before and during pandemic) with diagnosed COVID-19 in the family; when a member of the family was diagnosed with COVID-19, children tended to spend less time in PA.	Ghanamah and Eghbaria-Ghanamah (2021)
Japan	May 2020 and October 2020	Non-randomized experimental	78 children in school closure group & 113 controls	Self-report by youth	↓	The school closure group had more children who spent less time devoted to PA (56 %) compared to children in the non-school closure group.	Saito et al. (2021)
Portugal	March 23-April 1, 2020	Cross-sectional	2,159 children ages 6–12 years	Self-report by parent/caregiver	↓	There was a decrease in children’s PA. Girls played more without PA than boys during the pandemic. Children ages 6–9 years spent 21.95 % of their day in overall PA whereas children ages 10–12 years spent 16.32 % of their day in overall PA during the pandemic.	(Pombo et al., 2021)
Serbia	May 29-June 6, 2020	Cross-sectional	450 children ages 7–15 years	Self-report by parent/caregiver	↓	There was a small decrease in the number of PA participants. 63.4 % of children did exercise at home during the pandemic. 68 % of children exercised at home for < 30 min during the pandemic. During the pandemic, 69 % of parents considered their child’s PA level to be only enough to maintain physical form partially.	(Vuković et al., 2021)
Spain	September-December 2019 and March-April 2020	Cohort	291 children	Wrist-worn Actigraph accelerometer prior to pandemic Self-report by youth	↓	PA decreased (-91 +/- 55 min/day). 95.2 % of children worsened their PA during the pandemic. There were no sex, school, or weight status differences in changes in PA. Children with outside space at home or other big spaces (garage, attic, gym) , had less of a decrease in PA compared to peers without them.	(Medrano et al., 2020)
Tunisia	April 25-May 20, 2020	Cross-sectional	100 children ages 5–12 years	Self-report by youth and parent/caregiver	↓	PA decreased. Total PA decreased by 7 % for boys and 17 % for girls from before to during the pandemic.	(Abid et al., 2021)
Turkey	Not stated	Qualitative cross-sectional	10 children with autism spectrum disorders	Self-report by parent/caregiver	↓	Three major themes: possible benefits of PA during the pandemic, PA barriers during the pandemic, and solution suggestions for PA during the pandemic.	Esentürk (2021)
United Kingdom	July 14-August 14, 2020	Cross-sectional	927 children ages 5–11 years	Self-report by parent/caregiver	↓	Children spent less time in daily PA. The proportion of children with low PA (<30 min) increased from 3.7 % to 16.2 %. The proportion of children engaging in PA between 1.5 and 2 h and > 3 h reduced from 20.3 % to 13.5 % and from 10.1 % to 5.8 %, respectively.	Morgul et al. (2020)
U.S.	April 23-May 4, 2020	Cross-sectional	2,440 children	Self-report by physical education teachers, school administrators	↓	Time spent in PA and PE decreased. 78.8 % believed their students were obtaining either significantly less or somewhat less PA compared to their typical school day. For schools that closed their campuses, 86.1 % were reportedly able to deliver P.E. remotely. 79 % reported that students were accumulating either “significantly less” or “somewhat less” activity during the closure.	(Pavlovic et al., 2021)
U.S.	April 25-May 16, 2020	Cross-sectional	211 children ages 5–13 years	Self-report by parent/caregiver	↓	Parents perceived children’s PA had decreased. Average yesterday MET-min: 892 ± 902 Child age was significantly negatively associated with day-total MET-min of PA. Boys were more likely to participate in sports practice/training than girls during the pandemic. Children were more likely to perform PA at home or in the garage during the pandemic; children were less likely to perform PA on parks or trails during the pandemic.	[Anonymous, 2020]
U.S.	April-June 2020	Mixed-methods; cross-sectional	51 children ages 5–17 years	Self-report by parent/caregiver	↕	About half of parents said that children either maintained the same amount of activity, or in a few cases, increased their activity compared with before the pandemic. Many families went outside more frequently and for longer periods because of being at home and having a less structured school day. Parents attributed changes in activity to closure of school (recess, P.E. classes) and organized sports or dance.	(Neshteruk et al., 2021)
U.S.	March 2019-September 2020	Cohort	1,084 adolescents	Self-report by youth	↓	PA decreased; adolescents were less likely to report being physically active after the stay-at-home order.	(Chaffee et al., 2021)
U.S.	October 8-November 13, 2020	Cross-sectional	1,290 children ages 5–12	Self-report by parent/caregiver	↓	Parents of children receiving virtual instruction were more likely than were parents of children receiving in-person instruction to report that their child experienced decreased PA (62.9 % vs 30.3 %).Parents of children receiving combined instruction were also more likely than those of children with in-person instruction to report that their children experienced decreased PA (52.1 % vs 30.3 %).Parents of children receiving virtual instruction were more likely than parents of children receiving combined instruction that their children experienced decreased PA (62.9 % vs 52.1 %).	(Verlenden et al., 2021)

^a^Dates are listed as shown in the full-text article; some articles did not specify dates.

^b^There was not a defined age range for “children” or “adolescents”; whichever term(s) the authors used is listed.

^c^Age ranges are not listed if the information was not available in the full-text article.

^d^.↑indicatesanincreaseinPA,↓indicatesadecreaseinPA,↕indicatessomeincreaseandsomedecreaseinPA

MVPA = Moderate-to-vigorous physical activity.

*Note.* Articles are sorted alphabetically by country and chronologically by date of data collection.

### 3.1 Data collection timeframe and study design

**Fig. 2:**
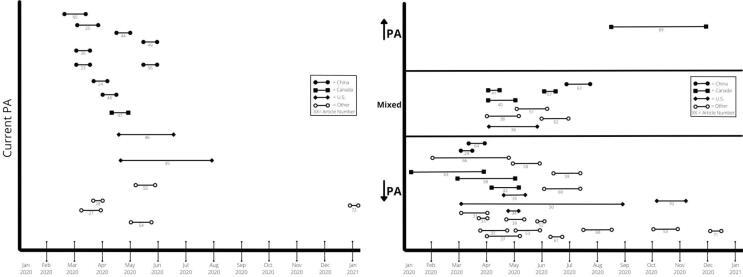
Comparisons of articles assessing current physical activity (PA) during the COVID-19 pandemic and change in PA from before to during the pandemic.

Data collection for most articles began in March 2020 (n = 10), (Kang et al., 2021, Zhang et al., 2020, Qin et al., 2021, Tso et al., 2022, Di Dalmazi et al., 2020, Pombo et al., 2020, Guo et al., 2021, Chaffee et al., 2021, Medrano et al., 2020, de Sá et al., 2020, Pombo et al., 2021) April 2020 (n = 15), (Pavlovic et al., 2021, Dunton et al., 2020, Neshteruk et al., 2021, Genin et al., 2021, Chambonniere et al., 2021, Abid et al., 2021, Mitra et al., 2020, Guerrero et al., 2020, Moore et al., 2020, Shuai et al., 2021, Ren et al., 2021, Alves et al., 2021:e12786., Alves et al., 2021, Tardif-Grenier et al., 2021, Xiao et al., 2021) May 2020 (n = 8), (Liu et al., 2021, Sciberras et al., 2022, Nathan et al., 2021, Vuković et al., 2021, Saito et al., 2022, López-Aymes et al., 2021, Parker et al., 2021, Lu et al., 2020) or June 2020 (n = 6). (Shepherd et al., 2021, Riazi et al., 2021, de Matos et al., 2020, Konstantinou et al., 2021, Hommes et al., 2021, Ng et al., 2021) The remainder of study data collection began in January 2020 (n = 2), (Hemphill et al., 2020, Xiang et al., 2020) February 2020 (n = 2), (Liu et al., 2021, Kouis et al., 2021) July 2020 (n = 2), (Zhu et al., 2021, Morgul et al., 2020) September 2020 (n = 1), (Pelletier et al., 2021) October 2020 (n = 1), (Verlenden et al., 2021) December 2020 (n = 1), (Ghanamah and Eghbaria-Ghanamah, 2021) or January 2021 (n = 1). (Morres et al., 2021) One article did not indicate when data collection began. (Esentürk, 2021) Xiang et al. reported the earliest “during” pandemic (January 3–21, 2020; public health emergency in Shanghai) (Xiang et al., 2020) while Morres et al. reported the latest “during'' pandemic (mid-January - beginning February 2021; second lockdown in Greece). (Morres et al., 2021) Four articles also included data from before the pandemic to examine changes in youth PA levels. (Chaffee et al., 2021, Medrano et al., 2020, Hemphill et al., 2020, Kouis et al., 2021) See Fig. 2a and 2b for article timeline.

Regarding study design, five articles included qualitative data (López-Aymes et al., 2021, Shepherd et al., 2021, Riazi et al., 2021, Pelletier et al., 2021, Esentürk, 2021) while all others included quantitative data collection. Only three articles used an objective PA measure, which included a wrist-worn accelerometer, (Medrano et al., 2020) Fitbit Charge 2, (Hemphill et al., 2020) and EMBRACE smartwatch. (Kouis et al., 2021) The majority of articles used PA self-report measures: data were reported by youth only (n = 19), parent/caregiver only (n = 18), both youth and parent/caregiver (n = 8), by parent or youth depending on age (n = 3), PE teachers and/or school/district administrator (n = 1), and two articles did not specify who reported the data. Finally, only 43 % (n = 20) of quantitative articles used a validated PA measure.

### 3.2 Study sample characteristics and country origin

Eight articles were among unique populations sub-groups: youth prenatally exposed to gestational diabetes, (Alves et al., 2021) youth with overweight status, (Alves et al., 2021:e12786.) youth with obesity, (Neshteruk et al., 2021) youth with independent mobility, (Pelletier et al., 2021) high school athletes, (Shepherd et al., 2021) patients with ADHD, (Shuai et al., 2021) youth with asthma, (Kouis et al., 2021) and youth with autism spectrum disorders. (Esentürk, 2021).

Most articles were based in China (n = 13), (Kang et al., 2021, Zhang et al., 2020, Qin et al., 2021, Tso et al., 2022, Guo et al., 2021, Shuai et al., 2021, Ren et al., 2021, Xiao et al., 2021, Liu et al., 2021, Lu et al., 2020, Xiang et al., 2020, Liu et al., 2021, Zhu et al., 2021) followed by Canada (n = 8), (Mitra et al., 2020, Guerrero et al., 2020, Moore et al., 2020, Tardif-Grenier et al., 2021, Shepherd et al., 2021, Riazi et al., 2021, Hemphill et al., 2020, Pelletier et al., 2021) United States (U.S.; n = 7), (Chaffee et al., 2021, Pavlovic et al., 2021, Dunton et al., 2020, Neshteruk et al., 2021, Alves et al., 2021:e12786., Alves et al., 2021, Verlenden et al., 2021) Australia (n = 3), (Sciberras et al., 2022, Nathan et al., 2021, Parker et al., 2021) Spain (n = 2), (Medrano et al., 2020, López-Aymes et al., 2021) France (n = 2), (Genin et al., 2021, Chambonniere et al., 2021) and Portugal (n = 2). (Pombo et al., 2020, Pombo et al., 2021) Other countries had one article each (Italy, Brazil, Cyprus, Greece, Czechia, Germany, Greece, Israel, Japan, Serbia, Tunisia, Turkey, and United Kingdom). The majority of articles based in China assessed current PA (n = 10) and were completed by April 2020 (n = 9). Conversely, only one Canadian article and two in the U.S. assessed current PA and the remaining examined changes in PA from before to during the pandemic. Finally, only nine of the 51 included articles provided evidence of increases in youth PA, four of which were in Canada.

### 3.3 Current versus change in PA

Articles clustered into two categories based on their assessment of PA: (1) current PA during the pandemic and (2) change in PA from before the pandemic compared to during the pandemic. Of the 51 articles, 18 examined current PA during the pandemic and 33 examined PA changes with only four using data collected from before the pandemic (n = 29 used participant recall). Main findings for each article regarding current PA or change in PA are summarized in Table 1, Table 2. The majority of articles reported a decrease in PA during the pandemic as compared to before. Seven articles reported mixed results (e.g., both increases and decreases in PA) during the pandemic as compared to before. (Neshteruk et al., 2021, Chambonniere et al., 2021, Mitra et al., 2020, Guerrero et al., 2020, Nathan et al., 2021, Ng et al., 2021) For example, Nathan et al. concluded overall weekly minutes of total PA did not change during COVID-19, but increases in outdoor play and decreases in organized PA were observed. (Nathan et al., 2021) Only one article reported PA increases among their participants as a result of increased unstructured play. (Pelletier et al., 2021).

### 3.4 PA type and location

Seventeen articles discussed PA type or location. (Pombo et al., 2020, de Sá et al., 2020, Dunton et al., 2020, Neshteruk et al., 2021, Chambonniere et al., 2021, Mitra et al., 2020, Guerrero et al., 2020, Moore et al., 2020, Xiao et al., 2021, Liu et al., 2021, Nathan et al., 2021, Vuković et al., 2021, López-Aymes et al., 2021, Riazi et al., 2021, Konstantinou et al., 2021, Hommes et al., 2021, Pelletier et al., 2021) Although PA type and location are conceptually different, (Szeszulski et al., 2021) some articles described type and location together (e.g., outdoor play); therefore, results and implications are described together. Of the five studies discussing current PA during the pandemic, (Dunton et al., 2020, Guerrero et al., 2020, Xiao et al., 2021, Liu et al., 2021, López-Aymes et al., 2021) most described common PA types (e.g., free play/unstructured, sports practice, circuit training, walking/biking, outdoor play). Three articles specifically highlighted the importance of outdoor play in increasing child PA during the pandemic. (Guerrero et al., 2020, Liu et al., 2021, López-Aymes et al., 2021) Of the articles examining change in PA, thirteen assessed PA type or location. (Pombo et al., 2020, Guo et al., 2021, de Sá et al., 2020, Neshteruk et al., 2021, Chambonniere et al., 2021, Mitra et al., 2020, Guerrero et al., 2020, Moore et al., 2020, Nathan et al., 2021, Vuković et al., 2021, Riazi et al., 2021, Konstantinou et al., 2021, Hommes et al., 2021, Pelletier et al., 2021) The majority of articles reported decreases in youth PA, such as decreased sports participation, (Mitra et al., 2020, Moore et al., 2020, Hommes et al., 2021) school and free time, (Konstantinou et al., 2021) outdoor activities, (Mitra et al., 2020, Moore et al., 2020, Riazi et al., 2021) school-based PA, (de Sá et al., 2020, Riazi et al., 2021) home-based PA, (Vuković et al., 2021) and unstructured play. (Pelletier et al., 2021) Several articles cited a decline in PA due to lack of outdoor time, (Chambonniere et al., 2021, Guerrero et al., 2020) school time, (de Sá et al., 2020, Konstantinou et al., 2021) and structured activities. (Moore et al., 2020, Vuković et al., 2021, Riazi et al., 2021) Two articles reported increases in outdoor play (Mitra et al., 2020, Nathan et al., 2021) and several reported an increase in activity or maintenance of activity levels due to increased time outdoors. (Pombo et al., 2020, Liu et al., 2021, Nathan et al., 2021) Neshteruk et al. concluded some youth reported increased outdoor time while others decreased. (Neshteruk et al., 2021) Although Pombo et al. did not find PA differences for older youth with or without outdoor spaces, (Pombo et al., 2020) three articles concluded PA was more likely to decrease when youth had limited access to outdoor areas. (Guo et al., 2021, Neshteruk et al., 2021, Chambonniere et al., 2021) Chambonniere et al. also reported PA decreased among 35.2 % of youth who lived in rural areas, 46.7 % in suburban areas, and 47.9 % in urban areas. (Chambonniere et al., 2021) Families with less active youth cited limited ability to go outside due to safety concerns and lack of parental supervision. (Neshteruk et al., 2021, Moore et al., 2020) One strategy parents employed to increase youth PA was by redirecting their children from screen-based activities to outdoor time. (Chambonniere et al., 2021, Guerrero et al., 2020).

### 3.5 Age group differences

A total of 15 articles reported findings regarding age differences in PA. (Kang et al., 2021, Zhang et al., 2020, Di Dalmazi et al., 2020, Pombo et al., 2020, Guo et al., 2021, Medrano et al., 2020, de Sá et al., 2020, Pombo et al., 2021, Dunton et al., 2020, Chambonniere et al., 2021, Mitra et al., 2020, Moore et al., 2020, Xiao et al., 2021, de Matos et al., 2020, Zhu et al., 2021) One reported no difference in current PA between younger versus older youth (de Sá et al., 2020) and seven stated younger youth participated in more PA than older youth during the pandemic. (Di Dalmazi et al., 2020, Pombo et al., 2020, Guo et al., 2021, Pombo et al., 2021, Dunton et al., 2020, Moore et al., 2020, Xiao et al., 2021) On the contrary, two articles stated older youth participated in more PA than younger youth during the pandemic. (Kang et al., 2021, Zhang et al., 2020) Among articles examining change in PA, one article reported no age differences PA from before the pandemic to during (Medrano et al., 2020) and four reported younger youth lost less or gained more PA than older counterparts. (Chambonniere et al., 2021, Mitra et al., 2020, de Matos et al., 2020, Zhu et al., 2021).

### 3.6 Sex or gender differences

Fifteen articles assessed differences between sex or gender. (Kang et al., 2021, Zhang et al., 2020, Pombo et al., 2020, Guo et al., 2021, Medrano et al., 2020, Pombo et al., 2021, Dunton et al., 2020, Chambonniere et al., 2021, Abid et al., 2021, Tardif-Grenier et al., 2021, Xiao et al., 2021, de Matos et al., 2020, Hemphill et al., 2020, Zhu et al., 2021, Morgul et al., 2020) The following paragraphs use whichever terminology was employed in the respective article (i.e., boy/girl or male/female). Of the six articles examining current PA, three stated no differences between sex or gender (Pombo et al., 2020, Tardif-Grenier et al., 2021, Zhu et al., 2021) Of those with sex or gender differences, Xiao et al. found male participants engaged in more PA, (Xiao et al., 2021) Zhang et al. found girls participated in more PA (i.e., higher MET-min per week), (Zhang et al., 2020) and Kang et al. found boys engaged in more vigorous PA, but less moderate PA, than girls. (Kang et al., 2021) Of the six articles focused on PA change by sex or gender, two did not find any differences: Hemphill et al. used objective pre- and during-pandemic PA data from wrist-worn Fitbits and found no significant time-sex interactions, (Hemphill et al., 2020) whereas Medrano et al. found boys participated in more PA than girls before the pandemic, but no sex differences when examining PA change. (Medrano et al., 2020) Only one article reported that boys, compared to girls, had a smaller decrease in PA from before the pandemic to during the pandemic; (Abid et al., 2021) whereas, two articles reported that girls/females had a smaller decrease in PA than boys/males. (Guo et al., 2021, de Matos et al., 2020) Three articles investigated change in PA and reported sex or gender differences in current PA during the pandemic, rather than differences in PA change from before to during the pandemic: Dunton et al. reported no differences in day-total MET-min, but found boys were more likely to participate in sports practice or training than girls; (Dunton et al., 2020) Morgul et al. reported boys scored higher for meeting the WHO guidelines for PA compared to girls; (Morgul et al., 2020) and Pombo et al. found girls engaged in more unstructured PA than boys. (Pombo et al., 2021).

## 4 Discussion

The objective of this systematic review was to identify, evaluate, and synthesize the literature on the impact of the COVID-19 pandemic on youth PA and make the findings available to public health practitioners, researchers, and policy makers on local and national levels. Overall, studies reported a decrease in youth PA from before to during the pandemic with mixed results among different characteristics. The review also highlighted literature gaps and inconclusive findings with wide variation in methods used in empirical articles, creating a challenge for researchers and practitioners to directly compare study results and aggregate overall literature data for *meta*-analyses.

## 5 Implications for youth PA

### 5.1 Geographic location

Reported empirical articles including youth physical activity based on geographic location provide helpful comparisons between China, Canada, and the U.S. However, few or no findings among other countries limits larger global comparisons. Given varying transmission timing and subsequent preventive measures, longitudinal studies are required to examine ongoing youth PA changes as the pandemic continues. Opportunity for future research includes increased studies across geographic locations and assessment of differences in youth PA by country of origin, region, or population density (e.g., urban, suburban, rural). Cultural context of study measures also needs to be considered and aligned to allow for data aggregation analyses.

### 5.2 PA type and location

Significant changes were observed in youth PA in regards to type and location leading to overall decreased PA. Consistent with recommendations from the American Academy of Pediatrics, Centers for Disease Control and Prevention, and Shape America, youth’s potential in acquiring PA benefits based on the context of location includes a reliance on school-based opportunities such as PE, recess, and organized sports. (Academy, 2021, America, 2021, Centers for Disease Control and Prevention. Considerations for Classroom Physical Activity during COVID-19.;, 2020, Chin and Ludwig, 2013, Sallis et al., 2001) However, perceptions can vary in how much activity youth acquire due to being outside. Increased measures and monitoring of youth activity in different environments may assist in aligning these perceptions. (Ferdinand et al., 2012, Franzini et al., 2009).

### 5.3 Age

Findings suggest younger youth engaged in more PA (or experienced less of a decrease in PA) compared to older youth. These age differences may be due to less reliance on school- and sports-based programs and higher reported active free play among younger youth. This 'play' has more spontaneity and less reliance on sustaining a single activity. (Burdette and Whitaker, 2005) Coinciding with transition to full-time schooling, there is also an association between lower activity levels in both cross-sectional and longitudinal analyses of children from 5 to 5.5 years. Given over 80 % of kindergarten time is spent participating in sedentary activities, starting school may explain part of the reduction. (Taylor et al., 2013) Despite the increased protective factors free play seemed to have for younger youth during the pandemic, evidence demonstrates time spent in school is a protective factor for older youth from excessive weight gain. School protective factors may be due to the Structured Days Hypothesis, (Brazendale et al., 2017) which posits structured days—preplanned, segmented, and adult-supervised compulsory environment—prevent occurrence of negative health outcomes including unhealthy changes in body composition and fitness loss. Rather than engaging in free play, it seems older youth often have increased sedentary time, reduced PA engagement, displaced sleep patterns, and unhealthy dietary patterns on less structured days. (Weaver et al., 2018).

Age differences in PA during the pandemic may also be attributed to changes in active transportation. A longitudinal analysis indicated that as youth age, the likelihood of using active transportation to school increases, peaks at age 10 years, and then decreases. (Pabayo et al., 2011) Therefore, during pandemic-related school closures, older youth likely experienced more unhealthy behaviors (e.g., decreased PA and increased weight gain) than younger youth, which could be a result of reliance on structured days including active transportation to school and other school-based PA. Standardized age ranges and youth cultural roles (e.g., expectations of household, school, or work contributions by older youth) should also be considered in future research for data alignment as should correlations between screen time and remote learning.

### 5.4 Sex or gender

Reflective of previous literature demonstrating higher PA within boys, articles examining PA changes tended to find boys/males engaged in more PA than girls/females. These findings are consistent with a previous study which reported higher PA levels among boys than girls. (Telford et al., 2016, Tudor-Locke et al., 2009, Kahn et al., 2008, Corder et al., 2016) Pre-pandemic, this discrepancy was attributed to higher participation in organized sports by boys; (Vilhjalmsson and Kristjansdottir, 2003) while in-person organized sports may have been cancelled during pandemic-related closures, participation may have continued through online or remote services. (Dunton et al., 2020) Public health interventions should specifically focus on increasing girls' PA by determining barriers and facilitators for overall PA and organized sports participation.

### 5.5 Study design

Consistent with literature prior to the pandemic, (Corder et al., 2010, McGarty and Melville, 2018) mixed results in youth’s PA before and during the pandemic may be due to discrepancies in measures and in parents’ perceptions of their child’s PA levels. These discrepancies include variation in data collection timeframe and different PA outcomes, (e.g., daily MVPA minutes, time spent in specific activities) which cause difficulties in cross-article assessments and conducting *meta*-analyses. Standardized measures are needed, and study designs should not rely on self-reported PA alone. However, it should be noted researchers may have been limited in the ability to employ objective activity devices during the pandemic, such as loaning accelerometers, due to hygiene precautions and restrictions on in-person data collection. Standardized methods and measures, even if self-report, in future research may better inform PA changes among youth and assist in preparation to maintain or increase youth PA in future pandemics and other times of widespread distress and change.

### 5.6 Current PA versus change in PA

One particular measure varying across articles was current versus change in PA. Without a baseline, it is difficult to determine the extent which the pandemic exacerbated PA disparities or if PA actually increased or decreased. When pre-pandemic PA was assessed in the articles, researchers usually used retrospective recall and asked participants to compare the child’s current and pre-pandemic PA, likely rendering multiple biases. Moving forward, there is an opportunity for researchers and practitioners to collect baseline data.

## 6 Implications for PA research and practice

Future research directions include having aligned PA measures to increase reliability and validity of findings and allow for aggregated data analyses such as a *meta*-analysis. Given most of the included studies used self-report PA data, findings should be interpreted with caution. It should be noted, however, that self-report measures are used in the majority of PA research; therefore, this is a not a limitation specific to this review, but rather PA research. While self-report measures do have validity and reliability, one solution may be increased use of standard objective measures (e.g., steps, MVPA minutes), which are more accurate than self-report. (Corder et al., 2010, McGarty and Melville, 2018) Furthermore, if a subjective measure is needed (e.g., lack of funding, restrictions on in-person data collection), PA measurement standardization is recommended. Additionally, age group standardizing may also assist direct article comparison and data aggregation. An important consideration with term and measure standardization includes the challenge of different cultures across the globe; therefore, studies assessing interpretations of terms across languages may also be needed.

Future research opportunities include PA maintenance during school and sport programs cancellations, barriers and facilitators to girls and adolescent’s PA, and aligning terminology and measures to increase generalizability and reliability of studies, respectively. Furthermore, the pandemic widened the gap of mental and physical repercussions of less youth PA creating additional need for public health promotion and practice efforts within these areas.(Corder et al., 2010, McGarty and Melville, 2018, Meade, 2021, Mittal et al., 2020)Watson and Koontz posited that because youth sports faced serious problems prior to the pandemic including high costs, professionalization, decreased participation, and barriers to access, considerations should extend beyond a return to ‘normal’. (Watson and Koontz, 2021) This unexpected pause of youth sport participation during the pandemic provides an opportunity to not only guide youth sport return, but invest in programs and organizations improving youth sport access for PA.

### 6.1 Opportunities to re-envision return to sport and school PA

Restarting school-based PA and sport programs must also address unique challenges acquired and losses sustained during the pandemic by re-envisioning the changed developmental setting to address ongoing changes in youths’ mental, emotional, and social needs. The loss of sports and school PE during the pandemic may have created and exacerbated challenges with healthy weight, mental well-being, fitness levels, self-efficacy, and skill acquisition. (Carrel et al., 2007, Booker et al., 2015) Additionally, disparities in program and schedule offerings often exist due to budget cuts, which hinder opportunities for PA. Challenges to restarting sports include having enough participants, staying safe, sport changes, being physically ready, costs, and impacts to individuals and facilities. (Staley et al., 2021) Previous actionable recommendations include increasing PA access, trauma-informed coach training, emphasizing social connections, teaching coping skills, and increasing PA access. (Staley et al., 2021) These recommendations aim to situate youth to be the focus, beneficiaries, and authority in returning to sports and school-based PA while being cognizant of their mental, emotional, and social health as changes in PA due to the COVID-19 pandemic seem to have ongoing and long-term consequences. (Hasson, 2017) Public health programmatic and policy strategies should be geared towards evolving and ongoing PA promotion within school and sport settings (e.g., activity breaks, active curriculum, free streaming/remote activities and lessons). (Dunton et al., 2020, Piercy et al., 2015).

## 7 Strengths and limitations

The goal of the systematic review was to identify, evaluate, and summarize findings from relevant studies to be more accessible to researchers and decision makers rather than produce a single estimate of the effect of the pandemic on youth PA. The review had several strengths such as assessing a time period during the COVID-19 pandemic that included initial global responses and subsequent fluctuations in virus transmission and associated regulations. In addition, the findings from the review included parsing out youth PA differences by location, age, and sex or gender. Among limitations, the review was not comprehensive since it did not include articles in languages other than English and non-peer-reviewed literature. Our inclusion criteria resulted in 51 scientific articles available at the time of the review. Although our review represented 19 countries, the majority were based in China, Canada, and the U.S. As such, generalizability to other countries should not be inferred. Second, articles published after May 2021 were not included and thus the review may not capture future pandemic fluctuations (e.g., mutations, response). We also were unable to explore socio-demographic and socio-economic status variability due to wide reporting heterogeneity within the literature. Due to these variables having significantly impact youth PA (Hasson et al., 2022, Hasson, 2017), they should be explored in relation to the COVID-19 pandemic in future research.

## 8 Conclusion

This systematic review synthesized available literature on the relation between the COVID-19 pandemic and youth PA. Articles assessed both current PA during the pandemic and change in PA from before to during the pandemic. The review found overall PA decreased during the pandemic. Several articles reported differences in PA changes between sub-populations, with decreases among older youth and mixed results between sex or gender. Results also indicated an increase in outdoor and unstructured youth PA during the pandemic. Findings suggest school closures hindered PA due to high reliance on school- and sport-based PA. To strengthen the evidence shared, youth PA measures used in research and practice need to be aligned including those measures beyond self-report to increase reliability, validity, and aggregation of findings. Articles in languages other than English, non-peer-reviewed literature, published after May 2021, and socio-demographic and socio-economic status variability need to be included to allow for reference to data from many world countries not represented in this review and to capture pandemic and socio-demographic and socio-economic fluctuations. Public health programming and policy should focus on evolving and ongoing PA promotion among youth, particularly to address existing disparities (e.g., age, gender, income) likely exacerbated by the pandemic. Additionally, practitioners and policy makers could leverage this time to re-envision how we promote youth sports and PA in the context of mental and physical health moving forward.

## Declaration of Competing Interest

The authors declare that they have no known competing financial interests or personal relationships that could have appeared to influence the work reported in this paper.
